# North Pacific and Arctic marine traffic dataset (2015–2020)

**DOI:** 10.1016/j.dib.2022.108531

**Published:** 2022-08-08

**Authors:** Kelly Kapsar, Benjamin Sullender, Jianguo Liu, Aaron Poe

**Affiliations:** a Center for Systems Integration and Sustainability, Michigan State University, 1405 S Harrison Road, East Lansing, MI 48823, USA; b Kickstep Approaches, 4303 Needle Cir., Anchorage, AK 99508, USA; c Center for Systems Integration and Sustainability, Michigan State University, 1405 S Harrison Road, East Lansing, MI 48823, USA; d Alaska Conservation Foundation, 1227 W. 9th Ave., Suite 300, Anchorage, AK 99501, USA

**Keywords:** Arctic, Shipping, Vessel traffic, Automatic Identification System, Satellite AIS, Pacific, Aleutian Islands, Bering Strait, AIS, Automatic Identification System

## Abstract

In this paper, we present a spatially explicit dataset of monthly shipping intensity in the Pacific Arctic region from January 1, 2015 to December 31, 2020. We calculated shipping intensity based on Automatic Identification System (AIS) data, a type of GPS transmitter required by the International Maritime Organization on all ships over 300 gross tonnes on an international voyage, all cargo ships over 500 gross tonnes, and all passenger ships. We used AIS data received by the exactEarth satellite constellation (64 satellites as of 2020), ensuring spatial coverage regardless of national jurisdiction or remoteness. Our analytical approach converted raw AIS input into monthly raster and vector datasets, separated by vessel type. We first filtered raw AIS messages to remove spurious records and GPS errors, then joined remaining vessel positional records with static messages including descriptive attributes. We further categorized these messages into one of four general ship types (cargo; tanker; fishing; and other). For the vector dataset, we spatially intersected AIS messages with a hexagon (hex) grid and calculated the number of unique ships, the number of unique ships per day (summed over each month), and the average and standard deviation of the speed over ground. We calculated these values for each month for all vessels as well as vessels subdivided by ship type and for messages from vessels > 65 feet long and traveling > 10 knots. For the raster dataset, we created a series of spatially explicit daily vessel tracks according to unique voyages and aggregated tracks by ship type and month. We then created a raster grid and calculated the total length, in meters, of all vessel tracks within each raster cell. These monthly datasets provide a critical snapshot of dynamic commercial and natural systems in the Pacific Arctic region. Recent declines in sea ice have lengthened the duration of the shipping season and have expanded the spatial coverage of large vessel routes, from the Aleutian Islands through the Bering Strait and into the southern Chukchi Sea. As vessel traffic has increased, the social and natural systems of these regions have been increasingly exposed to the risks posed by large ships, including oil spills, underwater noise pollution, large cetacean ship-strikes, and discharges of pollutants. This dataset provides scientific researchers, regulatory managers, local community members, maritime industry representatives, and other decision makers with a quantitative means to evaluate the distribution and intensity of shipping across space and through time.

## Specifications Table

**Table:** 

Subject	Environmental Science – Nature and Landscape Conservation
Specific subject area	Arctic marine changes, marine spatial planning, environmental impacts, marine ecology, transportation
Type of data	Map (shapefile and raster formats) Table
How the data were acquired	Data were derived from satellite acquired position signals from shipboard Automatic Identification System transponders. Raw data signals were received and pre-processed from binary signals into a human-readable format by exactEarth. The authors then cleaned the data, eliminated spurious records, and processed the remaining data into monthly summaries of vessel activity, aggregated by ship type.
Data format	Analyzed Filtered
Parameters for data collection	Through exactEarth's constellation of satellite AIS receivers, we collected data from all ships transmitting AIS signals in the Pacific Arctic during the study period (2015/01/01–2020/12/31). AIS signals are mandatory on all cargo ships greater than 500 gross tonnes, ships greater than 300 gross tonnes on an international voyage, and all passenger ships [1].
Description of data collection	AIS transmissions were collected via satellite receivers operated by exactEarth, a publicly traded data services company delivering real-time global location-based maritime vessel tracking information utilizing patented satellite AIS detection technology. We acquired the data in tabular format from this company, cleaned the erroneous signals, anonymized identifying information to preserve privacy, and processed it into monthly vessel traffic intensity maps that can be used for further analysis of shipping patterns in the study area.
Data source location	• City/Town/Region: North Pacific and Arctic Oceans. • Country: USA, Russia, International. • Latitude and longitude (and GPS coordinates, if possible) for collected samples/data: Approximate bounding box: 160E, 50N to 145W, 74N.
Data accessibility	Repository name: National Science Foundation Arctic Data Center Data identification number: Direct URL to data: Hex data: https://arcticdata.io/catalog/view/doi%3A10.18739%2FA2XG9FC41 1km Data: https://arcticdata.io/catalog/view/doi%3A10.18739%2FA2SQ8QJ9S 10km Data: https://arcticdata.io/catalog/view/doi%3A10.18739%2FA2NZ80R4J 25km Data: https://arcticdata.io/catalog/view/doi%3A10.18739%2FA2J678Z16

## Value of the Data

• **Unique and comprehensive source of data for maritime shipping in the north Pacific and southern Arctic Oceans:** These data present the most comprehensive information available on the spatial and temporal patterns of shipping in an expansive study area (∼8,000,000 km^2^) across a broad time horizon (six full years). Because this data type is not yet publicly available on a wide scale, there are very few comparable data sources available. These data enable critical and timely analyses of previously unknown issues surrounding the expansion of vessel traffic into novel portions of the Arctic marine ecosystem. Recent declines in sea ice have allowed unprecedented commercial accessibility, but until now, little was known about where, when, what types, and how many vessels are traveling the Pacific Arctic.
• **Broad utility:** These data can be immediately added to maps, statistical models, or other spatial representations of marine plans, including ongoing Arctic vessel routing discussions at local, national, and international levels.
• **Applicable to a diverse audience:** Our data is relevant to a wide variety of groups including locals stakeholders (particularly indigenous communities), marine biologists, ecologists, transport and commerce analysts, resource managers, regulatory agencies, policymakers, diplomats, the maritime industry, and marine-focused advocacy and non-profit organizations.
• **Foundation for environmental analyses, human health impact assessments, and enhancing safety of life at sea:** The datasets presented in this paper can be expanded upon through incorporation in a wide variety of applications, including those related to marine spatial planning, anthropogenic impact assessments (e.g., underwater noise propagation, vessel debris and pollution), oil spill risk assessments, marine search and rescue asset allocation, and projections of future shipping under climate change.

## 1 Data Description

The datasets presented in this manuscript represent six years of vessel traffic in the north Pacific and southern Arctic Oceans collected through satellite-based Automatic Identification System (AIS) ship tracking technology. AIS transponders are required by the International Maritime Organization on all cargo ships greater than 500 gross tonnes, all ships greater than 300 gross tonnes on an international voyage, and all passenger vessels [1]. AIS data are not publicly available and were acquired from exactEarth (https://www.exactearth.com/). Although atmospheric conditions, deliberate anthropogenic interference, or technological issues may limit occasional AIS signals, AIS data are increasingly recognized as one of the best sources of information on the spatial and temporal distribution of maritime traffic worldwide [2,3].

**Fig. 1:**
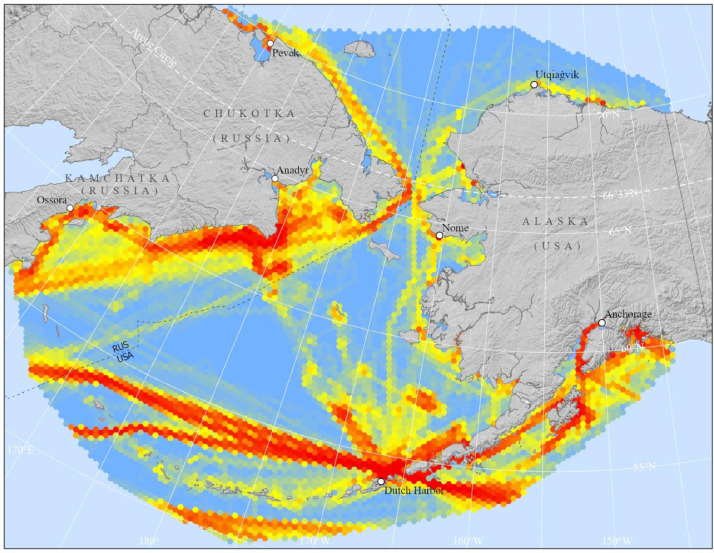
Map of study area boundaries with hex data depicting the relative number of unique maritime mobile service identities (MMSIs) identified within each hex in September of 2020. Cooler colors (blue) indicate fewer unique vessels while warmer colors (red) indicate more vessels.

The study area extends from Prince William Sound, through Cook Inlet and the Kodiak Island archipelago up through the Aleutian Islands north through the Bering and Chukchi Seas, past the northern edge of Wrangell Island and the western Beaufort Sea (approximately 51°-72°N). The study area spans the international dateline and includes both Russian and US exclusive economic zones (approximately 162°E to 145°W). Fig. 1 depicts the region across which AIS data were collected. The data begin in January of 2015, extend through December of 2020, and are temporally aggregated to a monthly scale.

We derived four independent data products from the AIS signals that are stored on the National Science Foundation's Arctic Data Center repository: a hexagon (hex) data set, a 10 km resolution raster data set, a 25 km resolution raster data set, and a 1-km resolution coastal raster data set (Table 1). All data products are projected from the original WGS 84 (EPSG 4326) to the Alaska Albers projection (EPSG 3338). To preserve anonymity and comply with AIS data licensing agreements, identifying attributes for all vessels have been removed and data have been aggregated from points into hex and raster cell values.

**Table 1:** Summary of marine vessel traffic data products.

	Resolution			File Type		
Format	Pixel/Hex Size (km)	Spatial Extent	Attributes	Type (by month and year)	By month and year	
Raster	1	Coastal (10km buffer)	Distance traveled (m)		✓	.tif
Raster	10	Entire study area	Distance traveled (m)	✓		.tif
Raster	25	Entire study area	Distance traveled (m)	✓		.tif
Hex	26.9*	Entire study area	Average Speed (knots)	✓	✓	.shp
			SD of Speed (knots)	✓	✓	
			Number of ships	✓	✓	
			Number of operating days	✓	✓	

⁎ This is an approximate calculation of the distance between the northern and southern edges of each hex.

Hex data are saved as 72 individual monthly shapefiles. Each hex has an area of 625 km^2^, resulting in an apothem of 13,432 m, or about 27 km from northern to southern edge. Individual hex cells contain five attributes: (1) a hexID that uniquely identifies each hex cell and is preserved across all hex files; (2) number of unique ships (as calculated by the total number of unique maritime mobile service identity (MMSI) numbers; (3) number of operating days (measures as the number of unique MMSI/date combinations); (4) average speed over ground (SOG) of all points within the hex; (5) standard deviation of the SOG. These five attributes are calculated for six subsets of data (cargo, tanker, fishing, other, all vessels, and long-fast vessels). See Hex Data Processing section below for a description of how these subsets and attributes were developed.

We developed raster datasets at three spatial resolutions, with varying subsets of data at each resolution (Table 1). The 1 km resolution coastal raster contains all vessel traffic within 10 km of the coastlines of the study area. The 10 km resolution dataset consists of 288 rasters containing values of total traffic subdivided into unique ship type, month, and year combinations (12 months x 6 years x 4 ship types = 288 files). Finally, the 25 km resolution raster dataset also contains the 288 rasters subdivided by ship type, month, and year. Unlike the hex data, the raster cells contain only one attribute, the total shipping traffic, measured as the total meters travelled for a given time step.

## 2 Experimental Design, Materials and Methods

### 2.1 General Data Processing

We received the data in tabular format after initial pre-processing from the original binary messages was completed by exactEarth. The tabular data were organized into daily comma separated value (csv) files with each row containing a single AIS message. In total, this amounted to 1462 individual csv files containing 1,169,510,073 AIS messages. To minimize memory usage and increase efficiency, we processed each month separately. All data were processed using R version 4.0.3 [4].

To clean the data, we first isolated position messages from static message types. While AIS signals come in one of 27 distinct message types [5], they fall into two primary groups. Position messages relay dynamic information from the vessel (e.g., navigational status, speed over ground, heading), while static messages convey information related to ship attributes (e.g., ship name, IMO number, draught, destination). Position messages are typically automatically derived by the AIS transponder and thus are not prone to human input error whereas static messages are manually input by shipboard AIS operators.

**Fig. 2:**
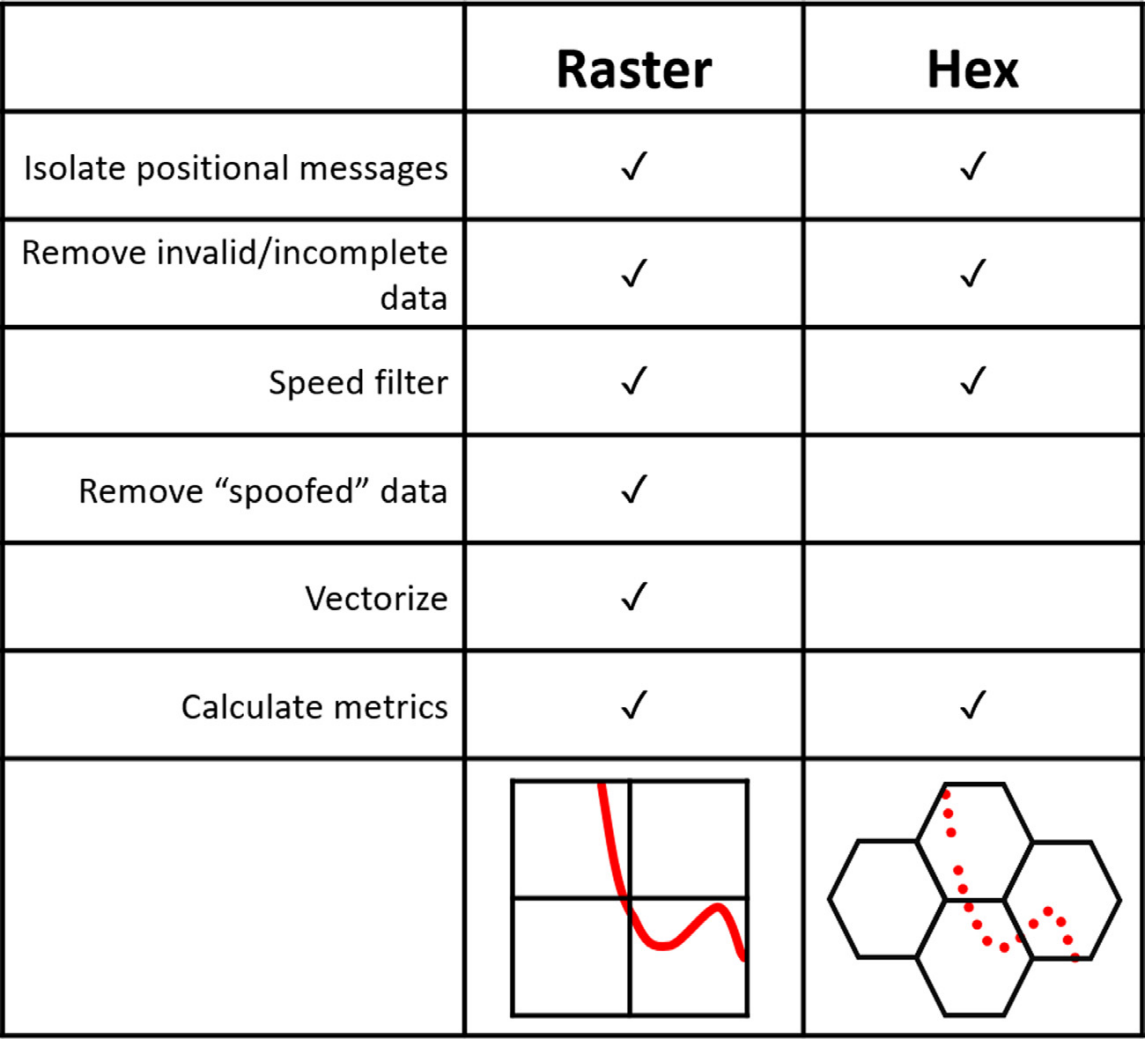
Data processing schematic for raster and hex grid datasets.

To process the data, we followed the steps laid out in Fig. 2. After isolating the position signals, we cleaned the data by removing messages with missing latitude or longitude values or messages with MMSI values less than eight digits. Large magnitude GPS errors are a relatively common occurrence in tracking datasets, but can be removed using a speed filter. We implemented a speed filter of 100 km/h (approximately 54 kn) using the Euclidean speed calculated between successive points. We assumed that points which would require vessels to travel faster than this speed are the result of GPS error and removed these points from the dataset.

Due to inconsistencies in data entry, we removed many of the static data attributes. We retained ship type, distance to bow, and distance to stern. Distance to bow and distance to stern can be used to derive ship length, which has been used as a proxy for ship size, a key correlate of lethal ship strikes to cetaceans [6]. We therefore retained this attribute to enable future calculations of ship strike risk. To calculate ship dimensions, we first removed all zero value entries from the distance to bow and distance to stern categories. We did this under the assumption that AIS transponders are typically placed on the bridge of a vessel and would thus have a non-zero distance to one of the vessels’ edges. Furthermore, these distance values are entered by operators and thus prone to manual entry errors. From the remaining values, we calculated the length of a vessel as the sum of the distance to bow and distance to stern.

Ship types are designated according to a 2-digit code transmitted in static messages. The first digit corresponds to a broad vessel category (e.g., 8 = Tanker), and the second digit corresponds to a specific vessel sub-category (e.g., 81 = Tanker, Hazardous category A). To minimize human error in ship type designation, we aggregated vessels into four broad categories: Cargo (AIS Type = 7X), Tanker (AIS Type = 8X), Fishing (AIS Type = 30), Other (all other ship types). After cleaning the data, we joined static messages to position messages using shared MMSI values.

### 2.2 Hex Data Processing

Hex grids are an increasingly prevalent geospatial data format. Hexagons have several advantages over traditional raster representations, including regularly and evenly distributed neighbors, the ability to store multiple attributes within a single geometry, and reduced distortions when projected at large scales [7]. We created a hex grid across our study area using the sf package version 0.9-8 in R version 4.0.3 [4,8]. We chose a hex cell size of 625 km^2^ (13,432 m apothem; approximately 27 km north to south) to align with our 25 km raster scale.

A key advantage of hex grids is the ability to store multiple attributed values within the same spatial unit. In our case, we created a unique identifier and calculated four metrics of shipping activity on six unique subsets of data for each hex cell (Table 2). We calculated hex cell values by spatially intersecting AIS messages with the hex grid. We then calculated all metrics by aggregating the values from all AIS messages within each hex.

**Table 2:** Description of hex data metrics.

Attribute	Units	Name in Shapefile	Description
Hex ID	NA	hexID	Unique ID number of each hex in the dataset. Hex IDs are preserved across all shapefiles.
Number of ships	vessels	nMMSI	Number of unique ship ID numbers (MMSIs) within each hex cell.
Number of operating days	operating days	nOprD	Number of unique operating days (MMSI/date combinations) within each hex cell.
Speed	knots	SOG	Average speed over ground (knots) within each hex cell.
Speed (Standard Deviation)	knots	SOGsd	Standard deviation of the speed over ground (knots) within each hex cell.

**Fig. 3:**
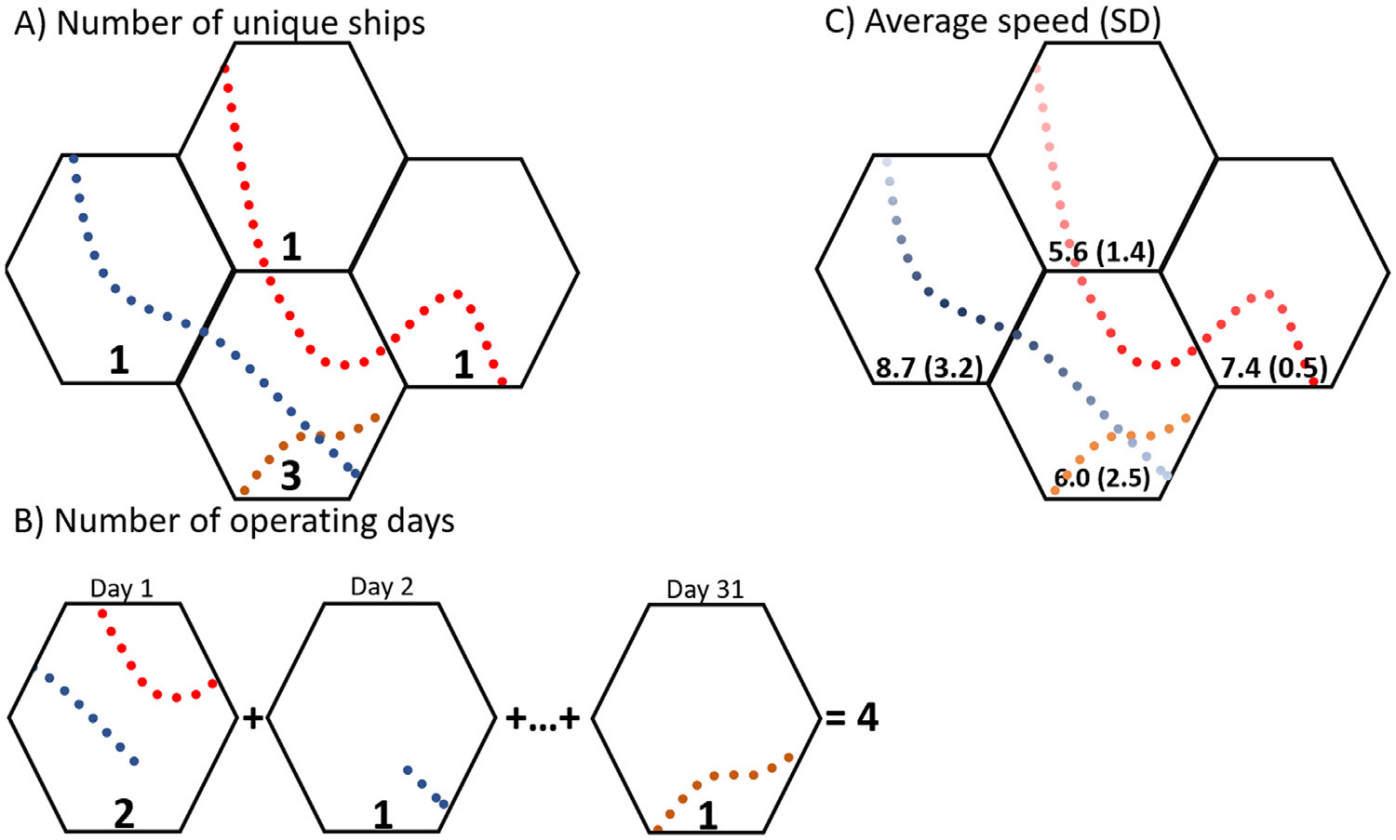
Schematic diagram explaining monthly hex data metrics. Dots represent individual Automatic Identification System (AIS) messages. Unique colors represent individual vessels. Subsets are (A) Number of unique vessels occurring within each hex in a given month; (B) Number of operating days (i.e., sum of the number of ships per day across a given month); (C) Average vessel speed in knots, and standard deviation of this speed value in parentheses. Darker colors represent higher average speeds.

We quantified the number of ships by calculating the total number of unique MMSIs present within the dataset in each month (Fig. 3A). MMSIs are permanent identifiers associated with unique vessels and are managed by the International Telecommunication Union [9].

While the number of unique ships per month gives a broad idea of the amount of traffic in a given area, it does not convey any information about residency time within a given region. For example, a single fishing vessel may occupy a given area for a week and that cell would only have a value of one unique ship. In order to capture a measure of vessel intensity within each cell, we developed a measure of operating days. We defined an operating day as the number of unique days one unique vessel occupied a given hex (Fig. 3B). In the above example, a fishing vessel working within one cell for the span of a week would have a value of seven operating days. If two vessels share the same cell for seven days, the number of unique ships would be two, but the number of operating days would be 14.

Depending on the purpose of the study, vessel speed can also be an important metric of the intensity of vessel traffic within a region. Faster vessel traffic is associated with increased underwater noise, elevated risk of ship strike to large cetaceans, and increased risk of collision with navigational hazards [6]. We calculated the average speed over ground (SOG) and the standard deviation of the SOG across AIS messages received within each hex within each month (Fig. 3C). While this metric does not present information about specific ships travelling at high speeds, it can be used to distinguish areas of transit (i.e., higher average speed) from ports and other areas of lingering vessel activity (i.e., lower average speed).

We designed these four metrics of vessel activity to be used either independently or in combination. For example, a hex with a small number of unique ships and a large number of operating days would indicate potential lingering activity, such as fishing. Furthermore, hexes with many vessels travelling at higher average speeds could be used to identify shipping routes.

To further parse out different spatial and temporal patterns of vessel activity, we calculated these metrics for six different subsets of data: all vessels (denoted with suffix “_A”), vessels categorized by ship type (cargo [_C], tanker [_T], fishing [_F], other [_O]), and long-fast ships (_L). For a description of how ship types were identified, see the section “General data processing” above. In the hex dataset, vessels with “NA” for ship type were predominately associated with non-ship entities (e.g., aids to navigation, terrestrial AIS receivers, floating platforms) and were removed from the dataset. Long-fast ships were defined as any ship over 65 feet in length with one or more AIS signal(s) with a SOG value greater than 10 kn in a given hex during a given month. Robust ecological models indicate that this class of vessel (> 65 feet and traveling > 10 kn) poses a significant risk of lethal injury to large cetaceans [10,11]. Average speed for long, fast ships was calculated as the average of all transmissions from a given hex from vessels over 65 feet in length and travelling at speeds greater than 10 kn within a given month.

### 2.3 Raster Data Processing

To capture the intensity of traffic within a pixel, we evaluated the total distance travelled by vessels within each cell in each month. This calculation required the addition of two extra processing steps not included in the hex data processing (Fig. 2). First, to calculate the distance travelled, we generated vessel tracks from the AIS point data. We did so by linearly interpolating between successive points (i.e., connecting the dots) at a daily timescale. Given the computational intensity of this process, this step was conducted on a high-performance supercomputer. Once daily transit segments had been created, we then spatially intersected all transit segments with raster grids of varying resolutions and calculated the total distance travelled by vessels within each pixel.

One challenge that we encountered after the data vectorization was the presence of vessel “spoofing”. AIS spoofing occurs when the position of a vessel is falsified or a vessel's identity is faked, either intentionally or by accident [12,13]. We identified several instances of spoofing in which two vessels were simultaneously transmitting AIS signals using the same MMSI number. When these signals were vectorized into daily track lines, this spoofing resulted in a zig-zag pattern as the AIS signals bounced back and forth between two indistinguishable ships. While spoofed lines were visually apparent in the vectorized data (e.g., large horizontal lines traversing hundreds of kilometers), they made up a very small portion of the total data set (283 spoofed lines removed across all six years of data). Given the rarity of spoofed track lines and the difficulties associated with separating the spoofed line into two daily transit segments, we chose to remove spoofed transit segments from the data set. We did so by implementing a length limit on daily transit segments. When vectorized, spoofed track lines resulted in very long daily transit segments that erroneously inflated the total distanced travelled within each pixel. To remove these spoofed lines from the dataset, we implemented a total daily distance travelled limit of 10,000 km. This 10,000 km value was chosen to be large enough that no ship could reasonably travel this distance within a 24 h period, and yet small enough that spoofed vessel track lines would result in daily transit segments longer than this distance threshold.

Once AIS data were vectorized and spoofed lines were removed, we rasterized the data at three different spatial scales (1, 10, and 25 km). The 10- and 25 km resolution raster datasets were chosen to represent the approximate spatial resolution of ecological data within the region. The 1 km resolution raster was calculated only for the coastal region surrounding the study area (identified by a 10 km buffer around all land area). The purpose of this coastal subset was to provide a high-resolution view of vessel traffic at a finer scale to evaluate potential interactions with both marine wildlife and human communities along the coast.

Similar to the hex dataset, we subset the raster data by month, year, and vessel type.

### 2.4 Data Omission

We used the number of unique ships (i.e., unique MMSIs) in the dataset to quantify rates of data removal during the data cleaning process for each month. Only a small percentage (0.29 ± 0.25%) of ships were removed for a lack of position information (i.e., static messages only). However, the largest number of vessels were removed due to invalid and/or incomplete MMSI numbers or latitude and longitude values (13.91 ± 8.12%). No unique ships were removed from the final data during the speed filtering step. Beyond this point in the data cleaning process, percentages of data removed differed slightly between the hex and raster data products due to differences in the data processing pipeline (Fig. 2). They are each discussed separately below.

For the speed hex data, another 13.81% of unique vessels were removed because they came from non-ship entities with an NA value for ship type (e.g., stationary platforms). Finally, 1.99 ± 1.05% of unique ships were removed because they were outside of the boundaries of the hex data (e.g., inland vessels). In total, this comes to a monthly average total of 30 ± 8.13% of unique MMSIs removed during the data cleaning process. While 30% of all unique ships may appear large, this only represents the removal of an average of 14.13 ± 2.53% of all AIS signals. This discrepancy between the larger percentage of unique ships removed and the smaller number of points removed is most likely due to data loss during the transmission to AIS satellite receivers resulting in a large number of unique vessels with very few, incomplete signals or from numerous non-ship entities infrequently transmitting AIS messages. In addition to these removals, during the data cleaning process 11.57 ± 3.24% of vessel width values, 10.98 ± 2.43% of vessel length values, and 0.28 ± 0.25% of speed over ground values were marked as NA.

For the raster datasets, an additional 11.99 ± 4.13% were removed for having less than three points or for failure to convert to a vector format. This error frequently occurred when ships transmitted AIS signals while in port. During this time, vessels were moving distances so small that they were beyond the granularity limits of the software, which resulted in a failure to interpolate between successive points. All combined, there was an average monthly removal of 26.19 ± 8.05% for raster data. However, given the large size of the study area, the six-year timeframe of the study, and the high transmission frequency of AIS (circa two minutes at maximum), these exclusions do not likely include a large number of unique vessels that were omitted from the analysis, but rather particular points within ship transits, data from non-ship platforms, or invalid signals (e.g., invalid MMSI).

### 2.5 Limitations and Considerations for Data Usage

While AIS data provide an unprecedented level of detailed information on vessel activities [3], several considerations must be taken into account when applying these data to an analysis. First, AIS data do not present a complete picture of all marine vessel traffic. Specifically, AIS transponders are not required on small vessels (< 300 gross tonnes) and thus many small vessels are excluded from this analysis. This gap in coverage may be particularly important in coastal areas and inland waterways and lakes around the world, where smaller craft are more likely to be located. If available, combining satellite-based AIS data with data from shore-based AIS receivers (which are more likely to pick up signals from less powerful AIS transponders) or other information on ship distribution (e.g., radar, port registries) could help augment these gaps in coverage, particularly in coastal waters.

Second, vessel type and vessel dimensions are subject to human error in the data entry process. Unlike positional messages which contain information derived directly from the vessel, static ship information is manually entered by AIS operators. In our dataset, this error can be seen in the large number of “other” ship types. It is thus important to note that when interpreting vessel activity, the total number of ships of a given type within a given area is likely a conservative estimate. Ships with AIS transponders disabled, small craft, and ships mislabeled as “other” all contribute to an undercount of the total number of vessels in an area.

Third, any increases in vessel activity should be interpreted with a degree of caution as two confounding factors could influence the interpretation of the number of ships within our study area: (1) increasing satellite coverage and (2) increasing adoption of AIS transponders. We discuss each of these factors and their potential influence on vessel activity metrics below.

**Fig. 4:**
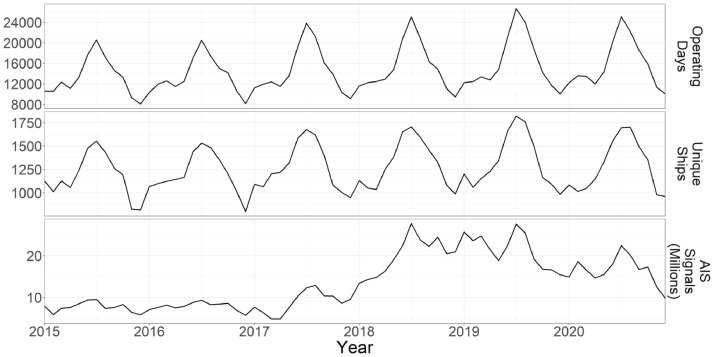
Comparison of the number of AIS signals, unique ships (i.e., MMSIs), and operating days (i.e., unique MMSI/date combinations) over the course of the study period.

As interest in AIS has grown over the past two decades, so too has the number of satellites with operational AIS receivers. During the study period, the number of satellites in the exactEarth constellation increased dramatically such that the number of satellite passes over our study area increased from approximately 70 per day in 2015 to over 700 per day in 2020 (with minor deviations in these numbers associated with maintenance and other activities). Concurrent with this increase in satellites, we see an increase in the total number of AIS signals received. However, an examination of the number of ships detected during this same period does not reveal a similar increase (Fig. 4). This discrepancy is most likely due to the fact that while the number of satellites did increase dramatically in 2018, the percent coverage (i.e., percentage of the day during which there was a satellite over the study area) only increased by approximately ∼10% (exactEarth, personal communication, 21 August 2021). Therefore, it is likely the case that entire ships did not go undetected early in the study period, however some AIS signals from those ships were lost due to limited satellite capacity to process all AIS signals. As the number of satellites increased, this rate of signal loss decreased leading to an increased number of AIS data points, but a relatively stable number of ships. While it is unlikely that a substantial number of vessels went undetected during the early portion of the study period that were later detected with the augmented satellite constellation, we advise caution when interpreting increases in the number of vessels across the entire study period.

Another factor to consider when interpreting increases in vessel activity over time is the increasing adoption of AIS transponders, particularly among smaller vessels. While large vessels (> 300 gross tonnes) on international voyages and passenger vessels were mandated to transmit AIS since approximately 2004 (depending on construction date), smaller vessels are not required to maintain operational AIS transponders. However, the advantages of AIS for safety of navigation are gaining recognition and more mariners are choosing to use AIS transponders. Without the incorporation of external information on the number of vessels using AIS transponders, it is difficult to distinguish between an increase in the number of vessels and an increase in AIS adoption rates within a given region. Examining shorter time periods (e.g., months) is one possible way to minimize the influence of AIS adoption rates on the total amounts of vessel traffic.

## Ethics Statements

These data were collected remotely via satellite by exactEartch Ltd (now Spire Global). As specified in exactEarth's privacy policy, we have de-identified all data and presented them in aggregated form to preserve individual vessel anonymity. These data do not contain information pertaining to human or animal subjects.

## CRediT authorship contribution statement

**Kelly Kapsar:** Software, Data curation, Methodology, Formal analysis, Visualization, Writing – original draft. **Benjamin Sullender:** Software, Data curation, Methodology, Formal analysis, Visualization, Writing – review & editing. **Jianguo Liu:** Supervision, Resources, Writing – review & editing. **Aaron Poe:** Project administration, Conceptualization, Funding acquisition, Resources, Supervision, Writing – review & editing.

## Declaration of Competing Interest

The authors declare that they have no known competing financial interests or personal relationships that could have appeared to influence the work reported in this paper.

## Data Availability

North Pacific and Arctic Marine Vessel Traffic Dataset (2015-2020) (Original data) (NSF Arctic Data Center).

## References

[1] IMO, (2002). https://wwwcdn.imo.org/localresources/en/KnowledgeCentre/IndexofIMOResolutions/AssemblyDocuments/A.917(22).pdf.

[2] Wright D., Janzen C., Bochenek R., Austin J., Page E. (2019). Marine observing applications using ais: Automatic Identification System. Front. Mar. Sci..

[3] Sullender B.K., Kapsar K., Poe A., Robards M. (2021). Spatial management measures alter vessel behavior in the aleutian archipelago. Front. Mar. Sci..

[4] R Core Team, R: A language and environment for statistical computing (2020) R Foundation for Statistical Computing, Vienna, Austria. https://www.R-project.org/.

[5] United States Coast Guard Navigation Center, AIS Messages, (2019). https://www.navcen.uscg.gov/ais-messages. Accessed October 16, 2021.

[6] van der Hoop J.M., Vanderlaan A.S.M., Cole T.V.N., Henry A.G., Hall L., Mase-guthrie B., Wimmer T., Moore M.J. (2015). Vessel strikes to large whales before and after the 2008 ship strike rule. Conserv. Lett..

[7] Sahr K. (2011). Hexagonal discrete global grid systems for geospatial computing. Cartogr. Remote Sens..

[8] Pebesma E. (2018). Simple features for R: standardized support for spatial vector data. R. J..

[9] International Telecommunication Union, Recommendation ITU-R M.585-9: Assignment and use of identities in the maritime mobile service, 2022. https://www.itu.int/dms_pubrec/itu-r/rec/m/R-REC-M.585-9-202205-I!!PDF-E.pdf.

[10] Conn P.B., Silber G.K. (2013). Vessel speed restrictions reduce risk of collision-related mortality for North Atlantic right whales. Ecosphere.

[11] Vanderlaan A.S.M., Taggart C.T. (2007). Vessel collisions with whales: the probability of lethal injury based on vessel speed. Mar. Mamm. Sci..

[12] K. Cutlip, Spoofing: one identity shared by multiple vessels, Global Fishing Watch. (2016). https://globalfishingwatch.org/data/spoofing-one-identity-shared-by-multiple-vessels/. Accessed October 11, 2021.

[13] Balduzzi M., Pasta A., Wilhoit K. (2014). Proceedings of the ACM International Conference Proceeding Series.

